# Financial Costs of Large Carnivore Translocations – Accounting for Conservation

**DOI:** 10.1371/journal.pone.0105042

**Published:** 2014-08-15

**Authors:** Florian J. Weise, Ken J. Stratford, Rudolf J. van Vuuren

**Affiliations:** 1 Research Department, N/a'an ku sê Research Programme, Windhoek, Khomas, Namibia; 2 Research Department, Ongava Research Centre, Outjo, Kunene, Namibia; University of Tasmania, Australia

## Abstract

Human-carnivore conflict continues to present a major conservation challenge around the world. Translocation of large carnivores is widely implemented but remains strongly debated, in part because of a lack of cost transparency. We report detailed translocation costs for three large carnivore species in Namibia and across different translocation scenarios. We consider the effect of various parameters and factors on costs and translocation success. Total translocation cost for 30 individuals in 22 events was $80,681 (US Dollars). Median translocation cost per individual was $2,393, and $2,669 per event. Median cost per cheetah was $2,760 (*n* = 23), and $2,108 per leopard (*n* = 6). One hyaena was translocated at a cost of $1,672. Tracking technology was the single biggest cost element (56%), followed by captive holding and feeding. Soft releases, prolonged captivity and orphaned individuals also increased case-specific costs. A substantial proportion (65.4%) of the total translocation cost was successfully recovered from public interest groups. Less than half the translocations were confirmed successes (44.4%, 3 unknown) with a strong species bias. Four leopards (66.7%) were successfully translocated but only eight of the 20 cheetahs (40.0%) with known outcome met these strict criteria. None of the five habituated cheetahs was translocated successfully, nor was the hyaena. We introduce the concept of Individual Conservation Cost (ICC) and define it as the cost of one successfully translocated individual adjusted by costs of unsuccessful events of the same species. The median ICC for cheetah was $6,898 and $3,140 for leopard. Translocations are costly, but we demonstrate that they are not inherently more expensive than other strategies currently employed in non-lethal carnivore conflict management. We conclude that translocation should be one available option for conserving large carnivores, but needs to be critically evaluated on a case-by-case basis.

## Introduction

The translocation of endangered animals, or those involved in human-wildlife conflicts, is a frequently used tool in conservation management. Translocation describes the deliberate movement of an organism from its source site to a recipient site that may either be within its extant or historic range (adapted from [1], [2]) or a novel but suitable environment. The objectives of translocations differ but commonly include population augmentation, introduction and re-introduction [3], or transfers into permanent captivity and population control. These interventions usually require substantial funding. Total costs for structured large carnivore re-introductions that involve translocations, for example, can be as high as hundreds of thousands [4] or millions of US Dollars (USD) [5], [6].

The use of translocation in the mitigation of human-carnivore conflicts and carnivore conservation in general has been viewed with skepticism [1], [7]–[15]. The strategy, though non-lethal and therefore publicly appealing [9]–[11], is controversial. Different concerns have been examined extensively and include large post-release movements [13], [15], reduced survivorship [10], [13], [16], possible creation of conflict at the recipient site [16] and quickly recurring conflict at the source site [8], [14]. Some translocations have in fact exacerbated human-carnivore conflicts, resulting in increased human mortality [7]. In addition to these problems, criticism points to a serious lack of cost transparency [1]. One assessment [17] found that only 3% of 180 animal translocation studies contained any cost data. Although only few attempts at cost reporting have been published thus far (e.g. [4], [18]–[21]), translocations are generally regarded as an “expensive” tool [2], [10], [22], [23]. From the little evidence available, it has been estimated that felids are amongst the most expensive large carnivores to translocate, with figures ranging up to $4,000 per animal [1]. Case-specific costs may even be higher in cases of long-term rehabilitation [19], [20]. This is highly relevant when considering that 71% of all translocated conflict carnivores are felids and that funding is usually very restricted [1].

In Namibia, landowner conflicts with large carnivores remain one of the major conservation challenges because the main conflict species, i.e. endangered cheetah (*Acinonyx jubatus*) and leopard (*Panthera pardus*), occur in large numbers outside of formally protected areas on livestock production ranches [24]–[27]. Because of persistent conflict with these species, Namibian farmers frequently request removal of individual predators (FJW unpublished data). Landowner attitudes towards and tolerance of large carnivores are unlikely to change if conflicts are not properly addressed.

The question arises whether translocation is an efficient use of conservation and management funding [10] because economic considerations play an important role in wildlife management and prioritization of restricted financial resources for species protection is paramount [28]–[31]. Thus, researchers and managers are responsible for providing decision-makers with transparent and accurate costing of their activities to enable comprehensive evaluations. However, it is the general omission of costing that still impedes adequate assessment of translocation as a contemporary conservation management tool [17]. Moreover, it often seems unclear to whom translocation costs should be attributed when a variety of different public and private stakeholders are involved in the process and have an interest in its successful outcome [11].

Here we report the financial cost of large carnivore translocations in Namibia across three species and comprising different translocation scenarios. The objectives of translocations in this study were to return perceived conflict predators into free-ranging environments with minimum potential for post-release conflict, and to enable their contribution to the wild gene pool. To our knowledge this is the first detailed documentation of case-specific large carnivore translocation costs. We identify the factors that influence translocation costs. Because of its public appeal (especially for charismatic large carnivores), we document which proportion of translocation costs we were able to raise from public support and did not accrue to the translocating agency. We assess translocation success and use this to derive a measure of conservation expenditure that we define as Individual Conservation Cost (ICC). This study neither argues for nor against large carnivore translocations – its' intention lies in the objective and transparent documentation of costs for consideration by wildlife managers.

## Methods

Cost data comprise information from translocations involving large carnivores that had been trapped under different circumstances on freehold properties in Namibia and that were perceived or confirmed as conflict predators. These animals were moved from the source site into a government-approved temporary captive facility with the purpose of subsequent release into free-range systems on private and public reserves. In some cases (when government permission could be obtained immediately) the animals were moved from source to recipient site without temporary holding. During temporary captivity the animals were maintained in enclosures that reflected their natural habitat and were in compliance with the national guidelines for the keeping of large carnivores in captivity [32]. Enclosures often had to be tailor-built for specific animals and necessitated electric fencing as well as reinforced materials for their safekeeping. Carnivores were kept with minimum human contact to avoid habituation to human presence and loss of natural fear. The animals were fed with horse, donkey or game meat. The length of temporary captivity varied considerably and was dependent on case-specific circumstances such as the sourcing of suitable recipient sites, manufacture of tracking devices, physical fitness, presence of dependent offspring, or the issuing of relevant permit documents. As a general rule, time in captivity was minimized as much as was practicable. Research was approved by the Ministry of Environment and Tourism in Namibia (permit number 1459/2008 with subsequent renewals), and all efforts were made to minimize stress and disturbance of study animals. Public and private landowners allowed access to their properties for research purposes.

Prior to translocation for release, carnivores were immobilized with combinations of Tiletamine and Zolazepam or Ketamine and ɑ2-agonists to carry out intensive health assessments and permanent marking, as well as fitting of monitoring technology. During immobilization, standard body measurements were recorded and biological samples collected. We fitted animals with Very High Frequency (VHF) radio transmitters or Global Positioning System (GPS) satellite transmitters at a ratio of <1.5% of their body weight to enable intensive post-release monitoring. Suitable transmitters were purchased from Advanced Telemetry Systems (Insanti, USA), Africa Wildlife Tracking (Pretoria, SA) and Sirtrack (Hastings, NZ). Immobilizations were reversed with appropriate antagonists (Atipamezole or Yohimbine).

We define transportation distance as the total linear distance from source to temporary captivity site and from there to the recipient site, or from source site directly to recipient site. We translocated carnivores at varying distances that were chosen to prevent homing to the original conflict site. Transportation distances were also influenced by the case- and species-specific availability of suitable recipient sites. During transportation the carnivores were not under the influence of any immobilizing agents and animals were moved in wooden or metal transport crates that allowed safe transportation whilst limiting the possibility of injury to the individuals.

In this study, candidate recipient areas had to meet the following criteria: i) be within extant range of the species; ii) contain appropriately documented and suitable predator guild and prey composition; and iii) present land uses with minimum potential for post-release conflict. Releases were carried out to resemble events of natural immigration of disperser animals into existing populations, i.e. animals were released as individuals or, in the case of cheetah, in small natural coalitions. Subsequent translocations into the same area were carried out at intervals of several months or years as opposed to continuous mass relocations (see [7]). At the recipient sites, carnivores were either hard released at permanent water sources (i.e. directly from the transport crate), or through the process of a soft release (after acclimatization to the local environment in a suitable enclosure) lasting from ten days to five months.

Reasons for translocations differ, and so do the definitions of desired outcomes and success (*cf.* [16], [21], [22], [33]). We define success by individual based on the following conditions: i) survival for 12 months post-release, ii) no significant livestock conflict (>5 units per year – the amount determined agreeable for compensation directly outside of release reserves), and iii) no homing to the source site. In contrast with others [15], [34], we do not consider site fidelity as a prerequisite for translocation success because carnivores were released into environments without predator-proof fencing, thus permitting free choice of movement. Similarly, reproductive success was not a condition for translocation success because despite intensive monitoring it is impractical to ascertain this with confidence for some individuals, especially males. We define Individual Conservation Cost (ICC) as the cost of successfully translocating an individual adjusted by the costs for unsuccessful translocations. We calculated ICCs by dividing the median cost for different translocation categories (i.e. individuals, each species and each release mode) by their respective success rates, thereby ensuring that both successful and unsuccessful cases contributed to the results.

We recorded translocation costs as true costs at the time when expenses occurred. All original costs were converted from South African Rand (ZAR) into USD to permit international comparisons. Conversions were made on the 15^th^ of every month during which costs occurred. Conversion rates ranged from $1.00 USD/9.86 ZAR to $1.00 USD/14.67 ZAR during the study. All values in this article are reported in USD unless otherwise is indicated. Any expenses contributing to the total cost were classified into one of the following distinct categories: i) government permits; ii) tracking (monitoring technology such as VHF and GPS transmitters as well as GPS data retrieval for the first 12 months); iii) veterinary expenses (salary, immobilization, identification tags, disease screening, health assessments, biological samples); iv) transport (travel from source site to temporary captivity site and subsequently to recipient site (or directly to release site) including fuel and standard vehicle wear-and-tear rates); v) captive holding (enclosure facilities); vi) captive feeding; and vii) staff salary. Although substantial, we exclude the cost of follow-up field monitoring of translocated carnivores from this analysis (although it was carried out) because its intensity, and consequently its cost, is highly biased towards the specific scientific objectives of the translocating agency and thus not representative of other translocation operations. Moreover, most carnivores in this study were fitted with GPS transmitters and could therefore be monitored remotely without significant extra costs other than the purchase of the tracking units.

We analyzed data using package ‘stats’ in R v. 3.1.0 [35]. Because cost data were not distributed normally and restricted to sample sizes of 30 or less, we utilized non-parametric statistics including a two-tailed Mann-Whitney U-Test (W) with a 95% confidence level for comparisons and Spearman's rank correlation coefficient (rs) to test associations between data sets. Results are presented as medians unless where comparisons are made with other studies. We recorded which cost elements were successfully funded externally through effective fundraising and we calculated overall proportions of total costs that could be reclaimed from public support. We assessed the effect of species, release mode, type of tracking technology, time spent in temporary captivity, degree of habituation, transportation distance and year of capture and release on translocation costs. We provide case-specific cost data in as much detail as possible (Table S1). Following others [36]–[39] we explored the data to look for associations between translocation factors and success. These included species, sex, age class, capture reason, captive time, transportation distance, post-release conflict, release mode, degree of habituation and cost. We initially used binary logistic regression to explore the data, and then more sophisticated non-parametric modelling approaches, including Bayesian Networks [38], [39], K-means cluster analysis [40] and Random Forests [41].

## Results

Cost and outcome data were recorded from 22 translocation events for a total of 30 animals −23 cheetahs, six leopards and one brown hyaena (*Hyaena brunnea*). Of these, nine cheetahs, all leopards and the hyaena were translocated as individuals, while the remaining 14 cheetahs were captured and released in groups (two groups of three, four groups of two). The animals had been trapped indiscriminately or deliberately on free-hold commercial farms in Namibia as suspected or confirmed livestock raiders during routine carnivore control operations by private landowners, or had been orphaned during these operations. One leopard had been confiscated from illegal captivity by the local wildlife authorities. The animals in this sample were trapped and translocated in the period 2008–2012 (see Table 1). We excluded the individual costs for 10 dependent sub-adult cheetahs that were translocated together with their mothers because the presence of offspring did not significantly increase case-specific costs for these females (cheetahs 07, 56, 58 and 59 in Table 1 respectively).

**Table 1:** Biological and technical details for 30 translocated large carnivores, 2008–2012.

Specimen	Age (years)	Sex	Year	Reason of Capture	Captivity (days)	Transportation Distance (km)	Tag Type	Habituation	Release Mode	Success	Total Cost (USD)	Comment
Aju001	2–3	F	2008	Indiscriminate	10	530	VHF	Wild	Hard – group	Yes	636.52	---
Aju002	2–3	F	2008	Indiscriminate	10	530	ID	Wild	Hard – group	Yes	443.52	Reproduced
Aju003	2–3	M	2008	Indiscriminate	10	530	VHF	Wild	Hard – group	No	636.52	Natural death
Pp006	4–5	M	2008	Livestock raider	16	372	VHF	Wild	Hard – single	Yes	867.97	Observed courtship
Aju007	7–9	F	2008	Livestock raider	61	461	VHF	Wild	Hard – single^a^	No	1,117.44	Recaptured
Pp015	4–6	F	2009	Confiscation	168	741	GPS	Wild	Hard – single	Yes	4,145.27	Reproduced
Aju017	5–7	M	2009	Livestock raider	175	493	GPS	Wild	Hard – single	Yes	3,980.34	Observed courtship
Aju018	5–7	F	2009	Livestock raider	157	473	GPS	Wild	Hard – single	No	3,994.94	Death from heat shock
Aju019	3–5	M	2009	Indiscriminate	62	314	VHF	Wild	Hard – group	No	829.78	Killed by hyaena
Aju020	6–8	F	2009	Indiscriminate	37	444	GPS	Wild	Hard – group	Unknown	4,228.91	Collar failure
Aju026	3–5	M	2009	Indiscriminate	12	184	GPS	Wild	Hard – single	Unknown	1,748.82	Collar failure
Pp027	2	F	2009	Orphan	639	316	GPS	Wild	Hard – single	Yes	3,027.05	Reproduced
Aju029	2–3	F	2010	Orphan	596	485	VHF	Semi-habituated	Soft – group	Yes	1,478.02	Reproduced
Aju030	2–3	M	2010	Orphan	446	467	GPS	Semi-habituated	Soft – group	Unknown	2,965.06	Collar failure
Aju034	3–4	M	2010	Indiscriminate	47	141	VHF	Wild	Hard – single	Yes	512.92	---
Aju038	3–4	M	2011	Indiscriminate	153	265	GPS	Wild	Soft – single	Yes	7,433.32	Observed courtship
Aju040	3–4	F	2012	Indiscriminate	1,184	348	GPS	Habituated	Soft – group	No	7,558.86	Killed by hyaena
Aju041	3–4	F	2012	Indiscriminate	1,184	348	VHF	Habituated	Soft – group	No	4,466.08	Shot
Aju042	3–4	M	2011	Orphan	1,055	824	VHF	Habituated	Soft – group	No	2,828.41	Shot
Aju043	3–4	M	2011	Orphan	1,106	842	GPS	Habituated	Soft – group	No	6,180.96	Shot
Aju044	3–4	M	2011	Orphan	1,055	824	VHF	Habituated	Soft – group	No	2,858.16	Shot
Pp045	2–4	M	2011	Livestock raider	206	764	GPS	Wild	Hard – single	No	2,005.67	Conflict behaviour - observed courtship
Pp047	2–4	M	2012	Livestock raider	183	400	GPS	Wild	Soft – single	Yes	2,208.40	---
Hbr055	1–2	F	2012	Livestock raider	3	63	GPS	Wild	Hard – single	No	1,671.82	Killed in vehicle accident
Aju056	5–7	F	2012	Indiscriminate	169	403	GPS	Wild	Hard – single^b^	Yes	3,848.38	Reproduced
Pp057	3–4	F	2012	Livestock raider	4	71	GPS	Wild	Hard – single	No	1,744.09	Killed in vehicle accident
Aju058	5–7	F	2012	Indiscriminate	260	411	GPS	Semi-habituated	Soft – single^a^	Yes	2,759.10	Reproduced
Aju059	4–6	F	2012	Indiscriminate	272	801	GPS	Semi-habituated	Soft – single^b^	No	2,577.36	Killed in gin trap
Aju065	6–7	M	2012	Indiscriminate	1	71	VHF	Wild	Hard – group	No	268.81	Homed to capture site
Aju066	6–7	M	2012	Indiscriminate	2	71	GPS	Wild	Hard – group	No	1,658.41	Homed to capture site

Aju indicates cheetah; Pp indicates leopard; Hbr indicates brown hyaena. Year is year of release. Indiscriminate captures include animals that predated on valuable game species. Semi-habituated  =  tolerance of human proximity only during feeding events before release. Habituated  =  tolerance of human proximity beyond feeding events before release. Group releases represent coalitions and not presence of offspring. Observed courtship  =  males seen in presence of wild females and displaying obvious courtship behaviour.

a translocated with 2 dependant offspring.

b translocated with 3 dependant offspring.

Carnivores were held for periods between 1-1,138 days, corresponding respectively to immediate release and the rearing of orphaned cubs. Transportation distances (collection - holding - release, or collection - release) were dependent on source site location and recipient site selection, but ranged from 63–842 km. Both hard (*n* = 19) and soft (*n* = 11) release modes were employed. Where possible GPS transmitters were deployed (*n* = 18) but for group releases of animals expected to remain together (only for cheetahs), we deployed a single GPS transmitter and then deployed VHF transmitters on the other animals. If GPS transmitters were not available within 3 months of capture, we also deployed VHF units for releases of individuals (*n* = 11 total VHF tracking units), but only in situations when an on-site post-release monitoring team was present at the recipient site. One identification collar was deployed on a cheetah female as part of a group release (Table 1).

**Figure 1:**
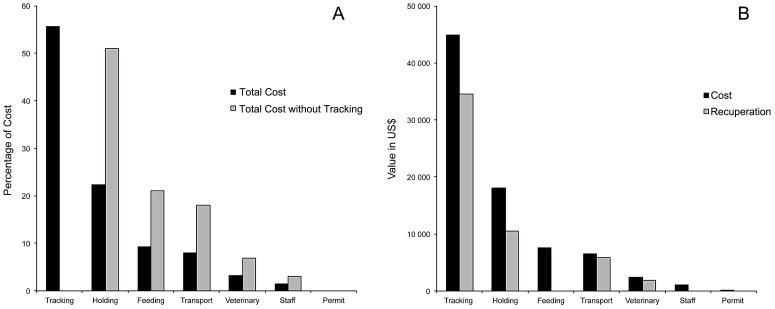
Analysis of large carnivore translocation costs. Panel A displays the distribution of total cost by category (black bars), demonstrating the impact of tracking technology (grey bars). Panel B compares cost and the amount recuperated from public interest groups in each category.

There was a wide range of total cost across animals, with the most expensive ($7,559) relating to an orphaned cheetah cub (held until old enough to release at 4 years, translocated 348 km and soft released with a GPS transmitter) and the cheapest ($269) associated with the immediate hard release of an adult cheetah translocated 71 km with a VHF transmitter (animals 40 and 65 respectively in Tables 1 and S1). The translocation of these 30 animals cost a total of $80,680.91, at a median cost per animal of $2,392.88, or $2,668.23 per translocation event (Table 2). The distribution of these costs into categories is shown in Figure 1a. Since the cost of equipment and expenses for GPS data retrievals comprises more than half of the total cost ($44,906.05, 56%), we also present a distribution of the costs without tracking in Figure 1a and Table 2. Each cost category showed a wide range of associated costs, although their magnitudes were very different. Permit costs were the cheapest ($1.27–$4.12), followed by staff ($13.77–$203.84), veterinary ($23.76–$756.22) and feeding ($0–$889.43) costs. Tracking ($27.00–$3,694.00) and holding ($0–$3,656.83) costs were the biggest factors in determining the total cost for an individual animal.

**Table 2:** Success rates and costs of large carnivore translocations in USD, including estimated Individual Conservation Cost (ICC) for each category.

		A – Total Cost				B – Total Cost without Tracking			
Total		80,680.91				35,774.86			
*Category*	Translocation Success Rate*	*Min*	*Median*	*Max*	*Estimated Cost per ICC*	*Min*	*Median*	*Max*	*Estimated Cost per ICC*
All individuals (*n* = 30)	0.44	268.81	2,392.88	7,558.86	**5,982.20**	75.03	627.31	4,275.83	**1,568.28**
All events (*n* = 22)	---	512.92	2,668.23	12,024.94	---	88.44	799.89	8,534.69	---
Cheetah (*n* = 23)	0.40	268.81	2,759.10	7,558.86	**6,897.75**	75.03	606.65	4,275.83	**1,516.63**
Leopard (*n* = 6)	0.67	867.97	2,107.03	4,145.27	**3,139.47**	160.71	675.16	1,641.81	**1,005.99**
Brown hyaena (*n* = 1)	0.00	1,671.82	---	1,671.82	---	88.44	---	88.44	---
Hard release (*n* = 19)	0.47	268.81	1,671.82	4,228.91	**3,557.06**	75.03	496.27	1,641.81	**1,055.89**
Soft release (*n* = 11)	0.40	1,478.02	2,858.16	7,558.86	**7,145.40**	905.07	2,638.16	4,275.83	**6,595.40**

A – Total cost for translocation study by category. B – Total cost without post-release tracking technology and expenses. *Individuals with unknown translocation outcome (*n* = 3) were removed from success rate calculations. An Individual Conservation Cost (ICC) is defined as the successful translocation in each category and accounts for failed attempts. Cost per ICC was estimated as the median cost/translocation success in each category, except for events because cheetah group releases resulted in both successful and unsuccessful translocations.

Total translocation cost and time spent in captivity were strongly correlated (rs = 0.654, *p*<0.001), but we found no correlation between total cost and transportation distance (rs = 0.199, *p* = 0.294), year of capture (rs = 0.203, *p* = 0.281) or year of release (rs = 0.280, *p* = 0.135). There was no significant difference in costs when analyzed by species, sex, age class and capture reason, although maximum individual costs were considerably higher for cheetah ($7,558.86; $4,275.83 without tracking costs) than for leopard ($4,145.27; $1,641.81 without tracking costs). We found a significant difference between costs by release method (W = 147.0, *p* = <0.02, *n* = 19 for hard release, *n* = 11 for soft release) and also found that, when tracking costs were removed, orphans (*n* = 6) cost significantly more than non-orphans (*n* = 24) (W = 147.0, *p* = 0.001).

**Figure 2:**
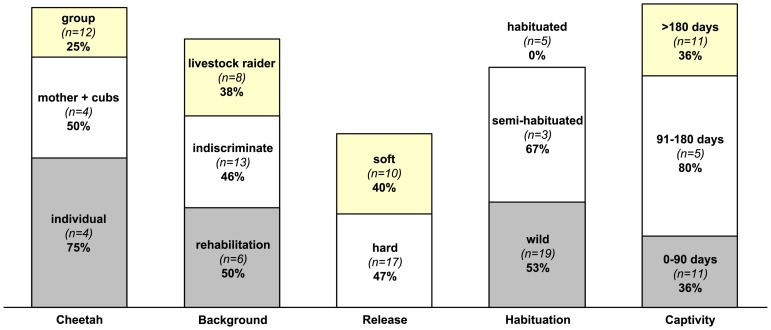
Large carnivore translocation success rates. Each column represents an analysis of all translocations for which outcome could be assessed (*n* = 27, for Cheetah *n* = 20) by different categories. Sub-categories show translocation success percentage, and are also scaled by the same factor. Rehabilitation in category Background includes confiscated leopard female Pp015.

We were able to evaluate translocation success for 27 of the 30 animals (Fig. 2). The GPS collars failed on three cheetahs (numbers 20, 26 and 30 in Table 1) before 12-months had elapsed. Four of the six leopards (67%) were classed as successful translocations, as were eight of the 20 cheetahs with known outcome (40%, see Tables 1 and 2). This gives an overall success rate of 44.4%. The ICC for cheetah was more than double that for leopard (Table 2). Of the 15 confirmed unsuccessful translocations, 11 died in their first year post-release (one leopard, nine cheetahs and the brown hyaena), two cheetahs homed back to the capture site (numbers 65 and 66), one cheetah was recaptured (number 07), and the remaining leopard (number 45) was reported to raid livestock.

Translocation success was significantly associated with degree of habituation (*p* = 0.023, Chi-2), as all five fully-habituated cheetahs were killed within 12 months of their release (Fig. 2). Four individuals were shot by landowners whilst the fifth was killed by a spotted hyaena (*Crocuta crocuta*). While we found no significant association between release mode and translocation success (*p* = 0.498, Chi-2), the five habituated cheetahs were all released from acclimatization bomas, significantly impacting on the soft release success rate. Translocation success was also not significantly associated with sex, age class or capture reason. Despite extensive exploration of the data set, we were unable to find any statistically significant multivariable models that described associations between costs, translocation success and the various translocation factors. Neither Generalized linear models (in our case binary logistic regression) nor more complex modelling approaches (see Methods) provided significant results. Figure 2 shows translocation success statistics by different categories and factors.

**Figure 3:**
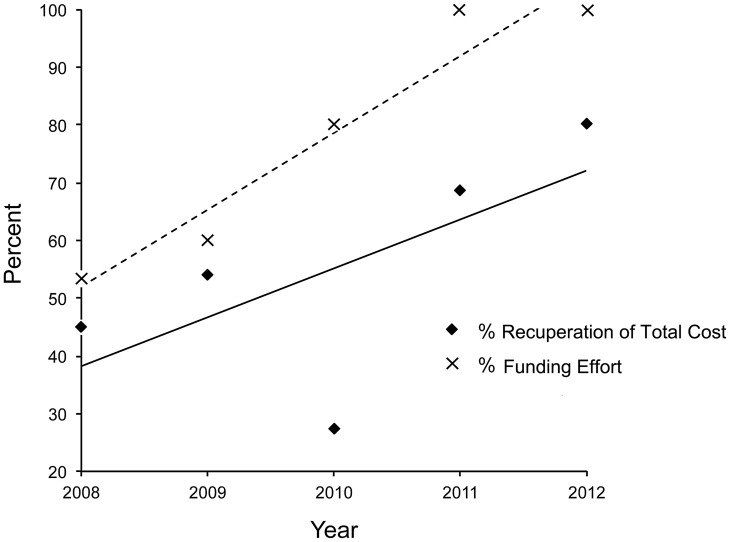
Percentage recuperation of total large carnivore translocation costs and funding effort across the study period. Year indicates year of release. Lines show best regression fits. The low recuperated value in 2010 was associated with a small sample of released individuals (*n* = 3) for which cost recuperation was less successful (see Table S1).

In this study we were able to recuperate costs from external funding sources for expenses in four categories – tracking, veterinary, transport and holding (see Fig. 1b and Table S1). In the case of tracking costs, the most costly category at 56% of total costs, external funding fully covered the costs of 20 of 29 transmitters deployed (15 of 18 GPS transmitters, five of 11 VHF transmitters) making a total recuperation of $34,517.62 (77%). Similarly we were able to recuperate 76% of veterinary costs, 91% of transport costs, and 58% of holding costs (Fig. 1b). The total amount recuperated from external sources was $52,791.58, equivalent to 65.4% of the total cost of all translocations. This figure decreases to 51.1% if tracking costs are removed. Total recuperation equates to an average of $1,762.45 per animal ($0–$6,270.42), or 65.5% of the average cost. Percentage of total costs recuperated per year significantly increased during the study and almost doubled from 2008 (45.2%) to 2012 (80.4%) as did our effort to source translocation funding – moving from eight proposals in 2008 to 15 proposals in 2012 (Fig. 3).

## Discussion

Conservation of large carnivores depends on the availability and most efficient allocation of financial resources. Carnivore managers in both the public and private sectors rely on using different approaches to deal with the manifold challenges of human-carnivore interactions – including compensation [42]–[47], translocation [16], [21], [33], [34], [48], [49], improved stock husbandry [50]–[62] and lethal control [49]. Translocation is a much implemented, yet largely under-studied strategy, especially with regard to its financial implications. We present the first report of detailed cost information with outcomes for translocations of three carnivore species in Namibia. We also introduce the concept of Individual Conservation Cost (ICC), which facilitates a simple and replicable evaluation of the cost-effectiveness of conservation effort.

In our study, translocation costs – across species, individuals and translocation scenarios – were highly variable (Tables 1 and S1) and success rates differed by species (Table 2). This variability reflects case-specific translocation circumstances (Fig. 2). Four leopards and eight cheetahs were translocated successfully without causing post-release conflict and effectively alleviating concern at the source sites for longer than 12 months. Our overall translocation success (44.4%) was very similar to that for other reported animal translocations (*cf.* [1], [17]). However, the translocation of leopards was nearly twice as successful as that of cheetahs whilst less than half as expensive - the ICC for the translocation of a leopard was $3,140 and that of a cheetah $6,898. Our cheetah translocation success was strongly influenced by the failed soft releases of five habituated individuals (animals 40–44). Therefore, the least successful translocations also involved the most expensive specimens (Tables 1 and S1), inflating the ICC for this species. We primarily attribute failure of these cheetahs to naivety resulting from habituation to humans during prolonged captivity [63]. Pre-release behavioural training has been shown to mitigate this vulnerability [19]. Long captive periods also had a strong effect on translocation cost - holding and feeding accounted for about 67% of the total cost of all translocations even after the primary cost factor (tracking technology) was removed (Fig. 1a). Our high costs for rehabilitations correspond with those for other long-term care cases of the same species [19], [20]. The limited release success of captive carnivores [2], [19], [63], [64] coupled with high costs for these subjects suggests that their use in translocations should generally be avoided.

Overall, tracking technology exerted the strongest influence on translocation costs (Fig. 1a). The deployment of tracking technology was considered important in this study, and carried out to enable intensive post-release monitoring for several reasons. Firstly, very little translocation-related ecological information was available for our focal species during the study period and existing records are based on small sample sizes [8], [15], [22], [48], [49], [65]. Secondly, the need for intensive post-translocation monitoring has been strongly emphasized in previous reviews of the strategy [2], [9]–[11], [17]. Lastly, we attempted to improve accountability of these translocations for local government and landowners. However, since tracking is not a biological prerequisite for translocation success, we argue that translocation costs could potentially be reduced by over 50% (Fig. 1a) or a significant proportion of these monitoring costs can be reclaimed from public support (Fig. 1b).

The use of soft releases for cheetahs resulted in case-specific costs exceeding $5,000 (Table 1). Despite higher costs (here resulting from the necessity to build large enclosures in remote semi-desert areas), soft release is reported to improve translocation outcomes for carnivores [10], [11], [16], [19], [37], [66]. Our pooled results show little beneficial effect of the technique in comparison with hard releases (Fig. 2) but our soft release success would increase to 80% if the five habituated cheetahs were excluded. Year of translocation showed no significant effect on total cost, indicating that inflation and currency conversions were not important factors in this study. We caution that the relative contribution of specific cost elements to the total (Fig. 1a) may be different in other parts of the world.

Public interest in and support of non-lethal carnivore management is indisputably growing and was an important component in this study. It cannot be denied that the charisma of large carnivores attracts substantial public funding support [67]. In Asia, international donor expenditures towards wild tiger conservation were estimated in excess of 40 million USD in less than one decade [68]. We successfully reclaimed a large amount of the total translocation cost from national and international non-governmental organizations, institutions and/or individuals (Fig. 1b). Support was also sourced through avenues such as in-kind donations of veterinary services and vehicles, direct funding of tracking technology (including data retrieval fees) and transport costs, logistical assistance at the recipient sites (e.g. soft release enclosures) and supportive tourism and volunteering enterprises. The proportion of costs we were able to reclaim increased during the study period (Fig. 3) but was directly linked to increasing efforts to source funding and thus resulted in considerable administrative efforts that need to be borne in mind by managers.

Although cost accounting has improved in recent years, direct comparison of our study with other non-lethal conflict mitigation alternatives remains difficult since there is no standardized methodology for reporting costs. Nonetheless, and even when considering ICCs for cheetah and leopard, translocations for conflict reduction or rehabilitation (*cf.* [19], [20]) were not more expensive than other conservation measures (Table 3) and should therefore not be rejected solely on cost grounds. Livestock compensation schemes, which provide an alternative symptomatic approach, frequently necessitate payments of hundreds of thousands or millions USD if they are implemented at large scales [42], [46], [69]. From a conservation perspective, investment into conflict prevention should take priority over symptomatic mitigation of damage [56], [57], [62]. For example, Namibian cheetahs can successfully be excluded from areas with valuable game species using cost-effective non-lethal swing gates [70]. Guard donkeys, guard dogs and thorn bomas have also been utilized to prevent livestock depredation by cheetahs and leopards [54]–[57]. The combined cost for acquisition and one year's maintenance of suitable guardian animals is consistently reported at less than $1,000 [50], [52], [53], [55], [58], [60], [61] and their use may decrease livestock losses by several thousands of USD per property per year [51], [56], [58], [59]. Furthermore, small-scale tourism ventures involving conflict carnivores have the potential to substantially outweigh financial losses from livestock depredation and significantly contribute to rural income generation. Monies earned from tracking conflict leopards exceeded losses twelve-fold [14]. Even though livestock protection is considerably more cost-effective than predator persecution [56], the availability of different conflict mitigation techniques has not stopped Namibian landowners from indiscriminately or purposefully trapping several hundred large carnivores every year (FJW unpublished data). Most of these animals, if not attended to by local wildlife authorities or non-governmental organizations, are still destroyed, resulting in large annual losses.

**Table 3:** Reported cost per carnivore (in USD) of different non-lethal carnivore conflict management options.

Country	Focal Carnivore	Method	Cost per Individual	Comment	Source
USA	Bear	Translocation	1,038	Cost estimate excluded staff salaries and administrative costs	Riley et al. 1994 [21]
Zimbabwe	Cheetah	Translocation	1,730	Removal of perceived conflict predator + re-introduction into protected area	Purchase 1998 [22]
n/a	Bears	Translocation	3,981	Mean value from translocation review	Fontúrbel and Simonetti 2011 [1]
n/a	Large felids	Translocation	3,941	Mean value from translocation review	Fontúrbel and Simonetti 2011 [1]
n/a	Canids	Translocation	2,875	Mean value from translocation review	Fontúrbel and Simonetti 2011 [1]
Namibia	Leopard	Translocation	2,334	Removal of perceived conflict predator	This study
Namibia	Brown hyaena	Translocation	1,672	Removal of perceived conflict predator	This study
Namibia	Cheetah	Translocation	2,827	Removal of perceived conflict predator	This study
South Africa	Cheetah	Translocation + Compensation	1,094^a^	Source landowner receives payment for not killing offending cheetah – recipient pays 50% of cost	Buk and Marnewick 2010 [18]
Italy	Wolf	Compensation	6,765^a^/year	Mitigation of livestock losses	Boitani et al. 2010 [43]
Russia	Leopard	Compensation	960^b^/year	Mitigation of livestock losses	Hötte and Bereznuk 2001 [44]
Kenya	Lion	Compensation	3,400/year	Mitigation of livestock losses	Maclennan et al. 2009 [45]
Sweden	Lynx/Wolverine	Performance payment	29,000	Payment per confirmed offspring to tolerant community to off-set expected lifetime damage	Zabel and Holm-Müller 2008 [47]

All values are rounded to the nearest US$. For comparison, all values are reported as means. We consulted a total of 57 publications that mentioned costs of non-lethal mitigation strategies. Here we report only those studies that measured cost using similar methodologies to our study.

a Where necessary, original values where converted from other currencies into USD on 16 April 2013.

b Annual cost extrapolated from monthly cost.

The significant ICC for translocations shows that this strategy should only be attempted in selected situations when the value of few individuals justifies such expenses (e.g. endangered species) and when these individuals enhance the population status of that species (*see* [4], [37]) by contributing to the free-ranging gene pool. High ICCs may only be justifiable in scenarios where the live-removal of individuals reduces landowner motivation to continue persecution and increases tolerance towards other free-ranging conspecifics. Appropriate candidate selection should therefore be the primary concern to maximize conservation output per translocation dollar spent.

## Supporting Information

Table S1 **Detailed cost breakdown per category for 30 translocated large carnivores (23 cheetahs, six leopards, one brown hyaena) in Namibia.** The table also indicates the amount of translocation cost recuperated from external funding sources. Data show a high degree of case-specific variability in terms of total cost per individual and cost recuperation.
(DOC)
