# Supplementary material for: Financial Costs of Large Carnivore Translocations – Accounting for Conservation
Source: PLoS One. 2014 Aug 15;9(8):e105042. doi: 10.1371/journal.pone.0105042 (PMC4134276; doi:10.1371/journal.pone.0105042)
Supplement: Table S1 — Detailed cost breakdown per category for 30 translocated large carnivores (23 cheetahs, six leopards, one brown hyaena) in Namibia. The table also indicates the amount of translocation cost recuperated from external funding sources. Data show a high degree of case-specific variability in terms of total cost per individual and cost recuperation. (DOC) [file pone.0105042.s001.doc]

**Table S1** – *Cost details in US$ per cost element for translocated large carnivores, 2008-2012.*

| **ID** | **Permit** | **Tracking** | **% Rec^a^** | **Veterinary** | **% Rec^a^** | **Transport** | **% Rec^a^** | **Staff** | **Holding** | **% Rec^a^** | **Feeding** | **Total** | **% Rec^a^** |
| --- | --- | --- | --- | --- | --- | --- | --- | --- | --- | --- | --- | --- | --- |
| Aju001 | 1.27 | 220.00 | 0 | 89.00 | 70 | 252.50 | 100 | 20.34 | 44.87 | 0 | 8.54 | **636.52** | **49.5** |
| Aju002 | 1.27 | 27.00 | 0 | 89.00 | 70 | 252.50 | 100 | 20.34 | 44.87 | 0 | 8.54 | **443.52** | **71.0** |
| Aju003 | 1.27 | 220.00 | 0 | 89.00 | 70 | 252.50 | 100 | 20.34 | 44.87 | 0 | 8.54 | **636.52** | **49.5** |
| Pp006 | 3.81 | 220.00 | 0 | 89.00 | 70 | 301.25 | 100 | 30.51 | 209.73 | 0 | 13.67 | **867.97** | **41.9** |
| Aju007 | 3.81 | 220.00 | 0 | 89.00 | 70 | 575.43 | 52 | 38.13 | 139.82 | 0 | 51.25 | **1,117.44** | **32.5** |
| Pp015 | 3.81 | 3,649.00 | 0 | 89.00 | 70 | 158.67 | 100 | 22.88 | 80.97 | 0 | 140.94 | **4,145.27** | **5.3** |
| Aju017 | 3.81 | 3,399.00 | 100 | 26.69 | 0 | 285.61 | 100 | 30.51 | 80.97 | 0 | 153.75 | **3,980.34** | **92.6** |
| Aju018 | 3.98 | 3,399.00 | 100 | 27.86 | 0 | 298.10 | 100 | 47.76 | 84.51 | 0 | 133.73 | **3,994.94** | **92.5** |
| Aju019 | 1.99 | 223.13 | 0 | 139.30 | 80 | 294.15 | 100 | 27.86 | 84.51 | 0 | 58.84 | **829.78** | **48.9** |
| Aju020 | 1.99 | 3,649.00 | 0 | 139.30 | 80 | 294.15 | 100 | 27.86 | 84.51 | 0 | 32.10 | **4,228.91** | **9.6** |
| Aju026 | 3.98 | 1,385.24 | 100 | 27.86 | 0 | 146.79 | 0 | 29.18 | 145.07 | 37 | 10.70 | **1,748.82** | **82.3** |
| Pp027 | 3.98 | 1,385.24 | 100 | 756.22 | 89 | 169.10 | 100 | 66.32 | 84.51 | 0 | 561.68 | **3,027.05** | **73.6** |
| Aju029 | 1.88 | 211.30 | 100 | 175.87 | 85 | 238.80 | 100 | 22.61 | 320.98 | 57 | 506.58 | **1,478.02** | **52.9** |
| Aju030 | 1.88 | 1,749.00 | 0 | 175.87 | 85 | 238.80 | 100 | 22.61 | 320.98 | 57 | 455.92 | **2,965.06** | **19.2** |
| Aju034 | 3.77 | 211.30 | 0 | 26.38 | 0 | 111.45 | 0 | 27.64 | 80.03 | 0 | 52.35 | **512.92** | **0.0** |
| Aju038 | 3.77 | 3,300.00 | 100 | 26.38 | 0 | 278.99 | 100 | 40.71 | 3,656.83 | 74 | 126.64 | **7,433.32** | **84.4** |
| Aju040 | 1.70 | 3,300.00 | 100 | 23.76 | 100 | 37.47 | 100 | 13.91 | 3,292.59 | 74 | 889.43 | **7,558.86** | **76.5** |
| Aju041 | 1.70 | 190.25 | 100 | 23.76 | 100 | 37.47 | 100 | 30.88 | 3,292.59 | 74 | 889.43 | **4,466.08** | **59.9** |
| Aju042 | 1.13 | 190.25 | 100 | 23.76 | 0 | 174.05 | 100 | 17.31 | 1,623.70 | 46 | 798.21 | **2,828.41** | **39.2** |
| Aju043 | 1.13 | 3,520.00 | 100 | 23.76 | 0 | 174.05 | 100 | 17.31 | 1,623.70 | 46 | 821.01 | **6,180.96** | **71.8** |
| Aju044 | 1.13 | 220.00 | 100 | 23.76 | 0 | 174.05 | 100 | 17.31 | 1,623.70 | 46 | 798.21 | **2,858.16** | **39.9** |
| Pp045 | 3.40 | 1,303.33 | 100 | 47.52 | 0 | 384.97 | 100 | 46.15 | 72.06 | 0 | 148.24 | **2,005.67** | **84.2** |
| Pp047 | 3.40 | 1,303.33 | 100 | 47.52 | 100 | 226.21 | 100 | 65.16 | 425.94 | 0 | 136.84 | **2,208.40** | **71.4** |
| Hbr055 | 4.12 | 1,583.38 | 100 | 28.86 | 100 | 35.67 | 100 | 19.79 | 0.00 | 0 | 0.00 | **1,671.82** | **98.6** |
| Aju056 | 4.12 | 3,300.00 | 100 | 28.86 | 100 | 173.16 | 100 | 39.58 | 150.28 | 37 | 152.38 | **3,848.38** | **92.4** |
| Pp057 | 4.12 | 1,583.38 | 100 | 28.86 | 100 | 64.67 | 100 | 59.37 | 0.00 | 0 | 3.69 | **1,744.09** | **96.1** |
| Aju058 | 4.12 | 1,583.38 | 100 | 28.86 | 100 | 340.89 | 100 | 203.84 | 333.88 | 56 | 264.13 | **2,759.10** | **77.6** |
| Aju059 | 4.12 | 1,583.38 | 100 | 28.86 | 100 | 451.25 | 100 | 54.70 | 150.28 | 37 | 304.77 | **2,577.36** | **82.2** |
| Aju065 | 2.06 | 193.78 | 100 | 28.86 | 100 | 29.42 | 100 | 13.77 | 0.00 | 0 | 0.92 | **268.81** | **93.8** |
| Aju066 | 2.06 | 1,583.38 | 100 | 28.86 | 100 | 29.42 | 100 | 13.77 | 0.00 | 0 | 0.92 | **1,658.41** | **99.0** |

^a^% Rec indicates percentage of cost recuperated from external funding sources. Aju indicates cheetah; Pp indicates leopard; Hbr indicates brown hyaena.
